# Supplementary material for: Combinatorial CAR design improves target restriction
Source: J Biol Chem. 2020 Dec 3;296:100116. doi: 10.1074/jbc.RA120.016234 (PMC7948400; doi:10.1074/jbc.RA120.016234)
Supplement: Figures S1–S8 [file mmc1.pdf]

Supporting Information

Combinatorial CAR design improves target restriction

Hakan Köksal[1], Pierre Dillard[1], Asta Juzeniene[2], Gunnar Kvalheim[1], Erlend B. Smeland[3,4], June H. Myklebust[3,4], Else Marit Inderberg[1], Sébastien Wälchli[1*]

[1]Department of Cellular Therapy, Department of Oncology, Oslo University Hospital, Oslo, Norway
[2]Department of Radiation Biology, Institute for Cancer Research, Oslo University Hospital, Oslo, Norway
[3]Department of Cancer Immunology, Institute for Cancer Research, Oslo University Hospital, Oslo, Norway
[4]K.G.Jebsen Centre for B cell malignancies, University of Oslo, Oslo, Norway

*Corresponding author: Sébastien Wälchli, Department of Cellular Therapy, Department of Oncology, Oslo University Hospital, Oslo, Norway. E-mail: sebastw@rr-research.no, Phone: +4722782314

List of Contents:

Supporting Figures S1-S8

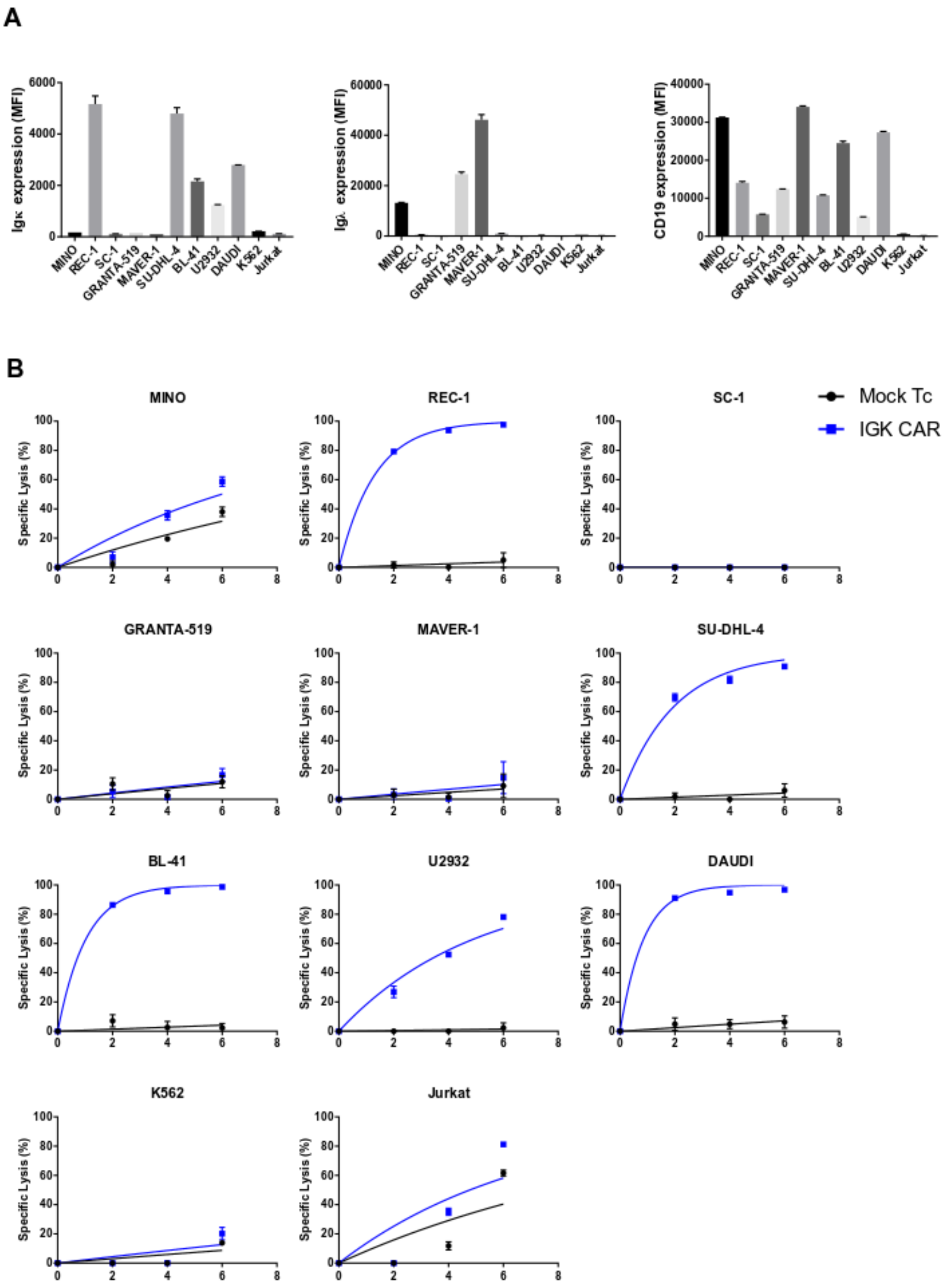

Figure S1: IGK CAR-expressing T cells target and specifically eliminate Igκ+ target cells. (A) Expression levels of Igκ, Igλ light chains and CD19 receptor for each cell lines. Data represent mean ± S.D. of duplicates. (B) BLI assay performed by Mock T cells and IGK CAR electroporated T cells at an E:T ratio of 25:1 for 6 hours. Data represent mean ± S.D. of quadruplicates. Representative data from one of two independent experiments are shown.

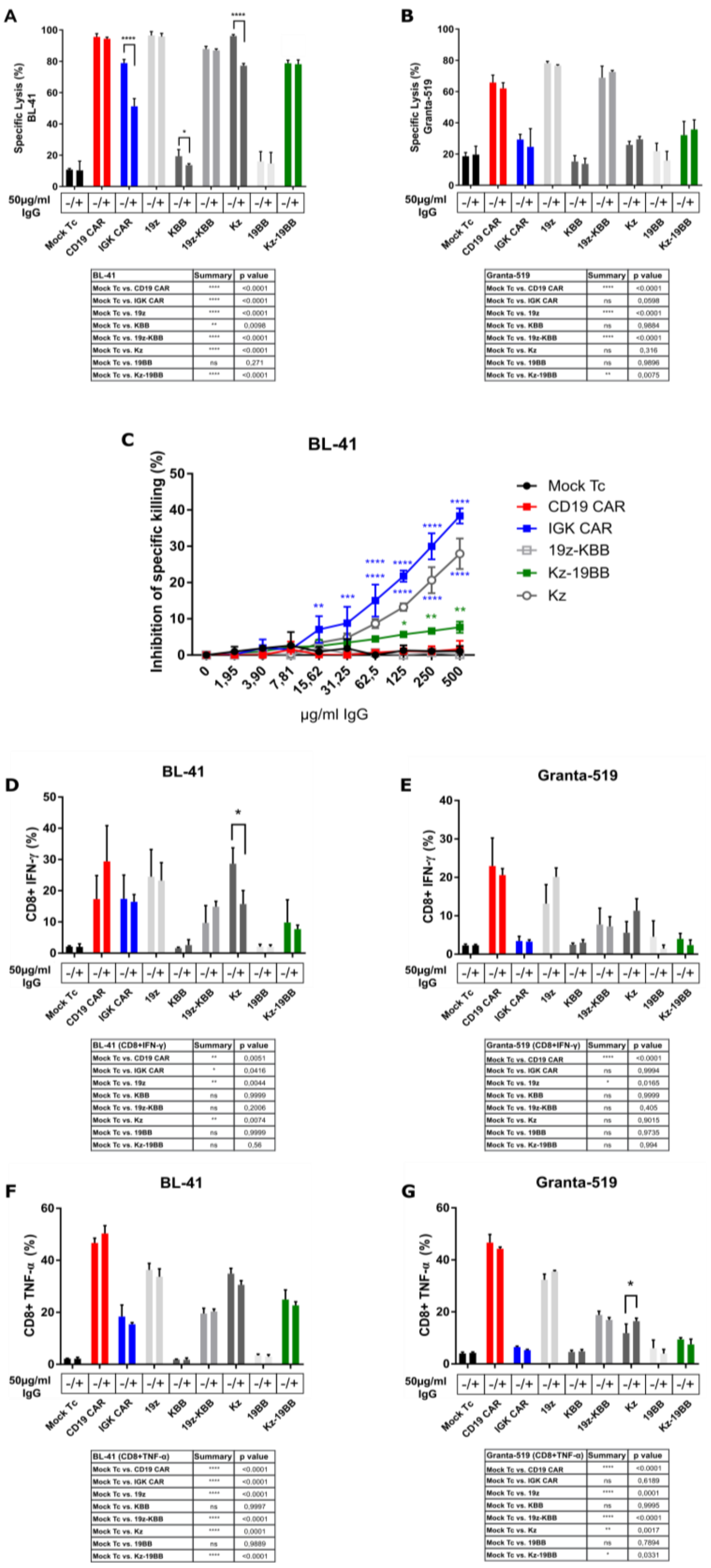

BL-41	Summary	p value
Mock Tc vs. CD19 CAR	****	<0.0001
Mock Tc vs. IGK CAR	****	<0.0001
Mock Tc vs. 19z	****	<0.0001
Mock Tc vs. KBB	**	0,0098
Mock Tc vs. 19z-KBB	****	<0.0001
Mock Tc vs. Kz	****	<0.0001
Mock Tc vs. 19BB	ns	0,271
Mock Tc vs. Kz-19BB	****	<0.0001

Granta-519	Summary	p value
Mock Tc vs. CD19 CAR	****	<0.0001
Mock Tc vs. IGK CAR	ns	0,0598
Mock Tc vs. 19z	****	<0.0001
Mock Tc vs. KBB	ns	0,9884
Mock Tc vs. 19z-KBB	****	<0.0001
Mock Tc vs. Kz	ns	0,316
Mock Tc vs. 19BB	ns	0,9896
Mock Tc vs. Kz-19BB	**	0,0075

BL-41 (CD8+IFN-γ)	Summary	p value
Mock Tc vs. CD19 CAR	**	0,0051
Mock Tc vs. IGK CAR	*	0,0416
Mock Tc vs. 19z	**	0,0044
Mock Tc vs. KBB	ns	0,9999
Mock Tc vs. 19z-KBB	ns	0,2006
Mock Tc vs. Kz	**	0,0074
Mock Tc vs. 19BB	ns	0,9999
Mock Tc vs. Kz-19BB	ns	0,56

Granta-519 (CD8+IFN-γ)	Summary	p value
Mock Tc vs. CD19 CAR	****	<0.0001
Mock Tc vs. IGK CAR	ns	0,9994
Mock Tc vs. 19z	*	0,0165
Mock Tc vs. KBB	ns	0,9999
Mock Tc vs. 19z-KBB	ns	0,405
Mock Tc vs. Kz	ns	0,9015
Mock Tc vs. 19BB	ns	0,9735
Mock Tc vs. Kz-19BB	ns	0,994

BL-41 (CD8+TNF-α)	Summary	p value
Mock Tc vs. CD19 CAR	****	<0.0001
Mock Tc vs. IGK CAR	****	<0.0001
Mock Tc vs. 19z	****	<0.0001
Mock Tc vs. KBB	ns	0,9997
Mock Tc vs. 19z-KBB	****	<0.0001
Mock Tc vs. Kz	****	0,0001
Mock Tc vs. 19BB	ns	0,9889
Mock Tc vs. Kz-19BB	****	<0.0001

Granta-519 (CD8+TNF-α)	Summary	p value
Mock Tc vs. CD19 CAR	****	<0.0001
Mock Tc vs. IGK CAR	ns	0,6189
Mock Tc vs. 19z	****	0,0001
Mock Tc vs. KBB	ns	0,9995
Mock Tc vs. 19z-KBB	****	<0.0001
Mock Tc vs. Kz	**	0,0017
Mock Tc vs. 19BB	ns	0,7894
Mock Tc vs. Kz-19BB	*	0,0331

Figure S2: Combinatorial CAR T cells are less sensitive to serum inhibition compared to individual components. (A-B) BLI killing assay of Mock T cells and CAR construct electroporated T cells co-cultured with Igκ^{+} BL-41 or Igκ^{-} Granta-519 cell lines for 10 hours (10:1 E:T ratio) in the presence or absence of serum purified IgG (50µg/ml). Data represent mean ± S.D. of quadruplicates. Representative data from one of three independent experiments are shown. (C) BLI killing assay of Mock T cells and CAR construct electroporated T cells co-cultured with BL-41 cell line for 10 hours in presence of increasing concentration of IgG (10:1 E:T ratio). Specific lysis inhibition corresponds to the difference of cytotoxic capacity of each construct between IgG+ and IgG- conditions. Data represent mean ± S.D. of triplicates. One-way ANOVA was performed between Mock T cell and other groups for each IgG concentration. (D-G) CD8+ T-cell intracellular cytokine staining (IFN- γ and TNF- α) after co-cultivation with BL-41 or Granta-519 cell lines in the presence or absence of IgG at 50µg/ml (for 24 hours, 1:2 E:T ratio) Data represent mean ± S.D. of triplicates. Representative data from one of two independent experiments are shown. (A,B,D-G) Significance was assessed by Student t-test comparing IgG+ and IgG- conditions and by one-way ANOVA between Mock T cells and any other group (IgG-). *P < 0.05, **P < 0.01, ***P < 0.001, ****P<0.0001.

A

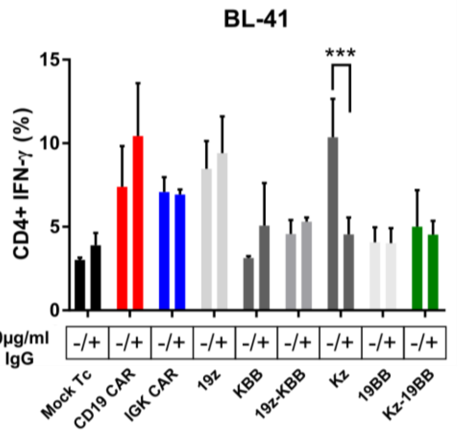

BL-41 (CD4+IFN-γ)	Summary	p value
Mock Tc vs. CD19 CAR	*	0,0189
Mock Tc vs. IGK CAR	ns	0,0614
Mock Tc vs. 19z	**	0,0033
Mock Tc vs. KBB	ns	0,9999
Mock Tc vs. 19z-KBB	ns	0,7514
Mock Tc vs. Kz	***	0,0002
Mock Tc vs. 19BB	ns	0,9483
Mock Tc vs. Kz-19BB	ns	0,5271

B

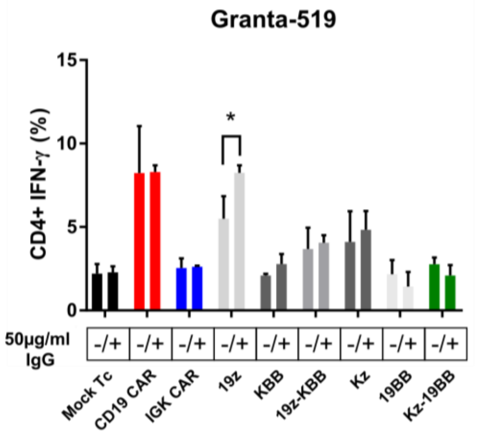

Granta-519 (CD4+IFN-γ)	Summary	p value
Mock Tc vs. CD19 CAR	***	0,0001
Mock Tc vs. IGK CAR	ns	0,9974
Mock Tc vs. 19z	***	0,0008
Mock Tc vs. KBB	ns	0,9996
Mock Tc vs. 19z-KBB	ns	0,2346
Mock Tc vs. Kz	ns	0,0716
Mock Tc vs. 19BB	ns	0,9894
Mock Tc vs. Kz-19BB	ns	0,9996

C

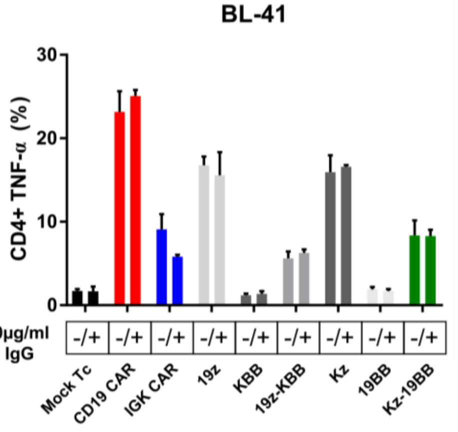

BL-41 (CD4+TNF-α)	Summary	p value
Mock Tc vs. CD19 CAR	****	<0.0001
Mock Tc vs. IGK CAR	**	0,0012
Mock Tc vs. 19z	****	<0.0001
Mock Tc vs. KBB	ns	0,9976
Mock Tc vs. 19z-KBB	**	0,0092
Mock Tc vs. Kz	****	<0.0001
Mock Tc vs. 19BB	ns	0,9999
Mock Tc vs. Kz-19BB	***	0,0004

D

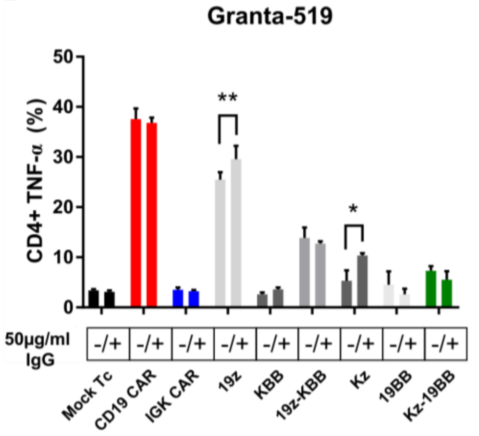

Granta-519 (CD4+TNF-α)	Summary	p value
Mock Tc vs. CD19 CAR	****	0,0001
Mock Tc vs. IGK CAR	ns	0,9999
Mock Tc vs. 19z	****	0,0001
Mock Tc vs. KBB	ns	0,9999
Mock Tc vs. 19z-KBB	**	0,0013
Mock Tc vs. Kz	ns	0,1182
Mock Tc vs. 19BB	ns	0,9997
Mock Tc vs. Kz-19BB	ns	0,3761

Figure S3: Combinatorial CARs limit serum inhibition while preserving CD3ζ driven specificity. (A-D) CD4+ T-cells intracellular cytokine staining (IFN- γ and TNF- α) after co-cultivation with BL-41 or Granta-519 cell lines in the presence or absence of IgG (50µg/ml) (for 24 hours ,1:2 E:T ratio) Data represent mean ± S.D. of triplicates. Representative data from one of two independent experiments are shown.

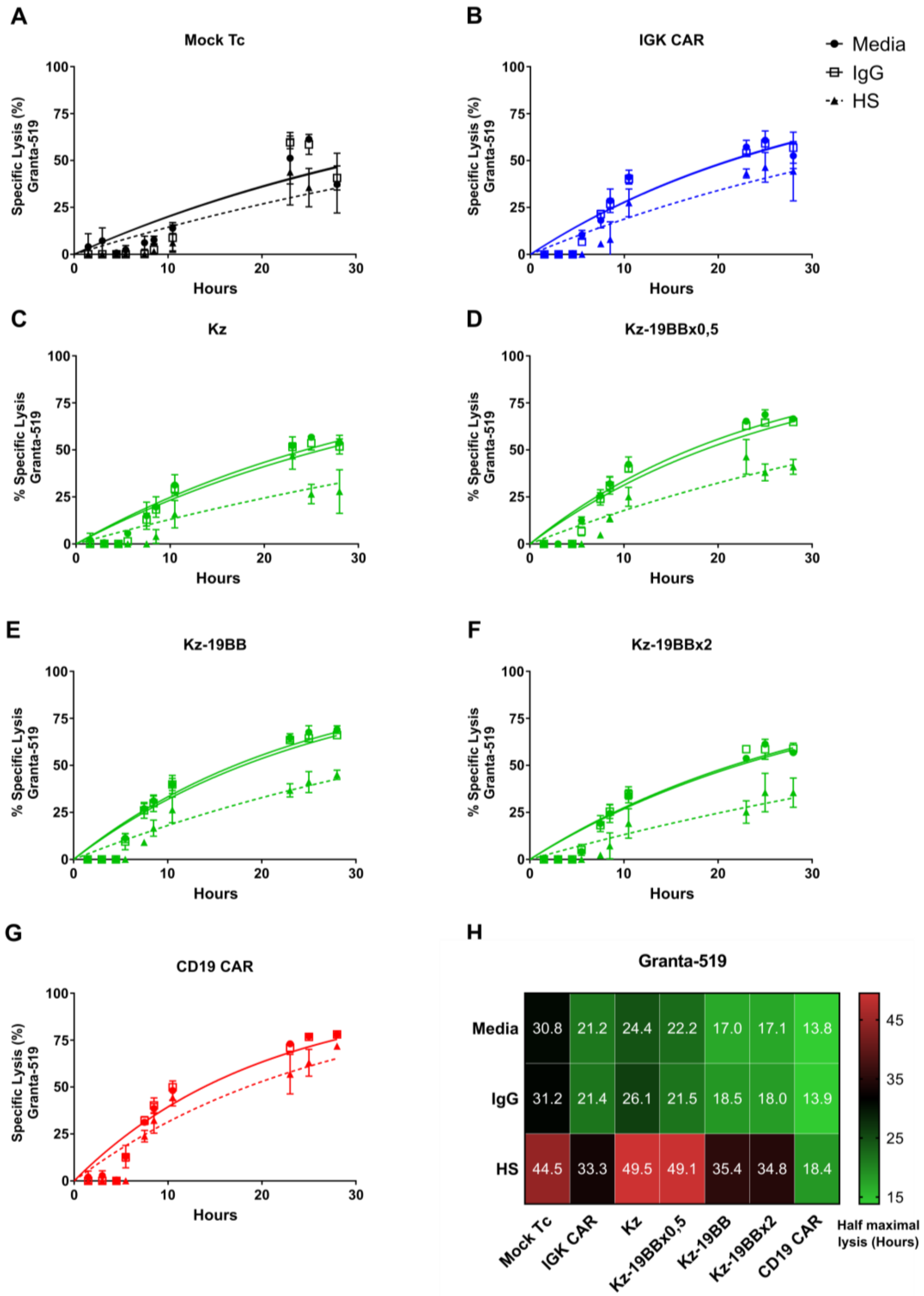

Figure S4: Kz-19BB expressing T cells also target CD19+ cells with each increased concentration of 19BB part. (A-G) BLI killing assay of Mock and CARs construct mRNA electroporated T cells co-cultured with Granta-519 cell lines either in presence or absence of IgG (50µg/ml) or 25% human serum. H) Heat maps of half maximal lysis values obtained via one phase exponential fitting against Granta-519. Data represent mean ± S.D. of quadruplicates. Representative data from one of three independent experiments are shown. **P* < 0.05, ***P* < 0.01, ****P* < 0.001, ****P<0.0001.

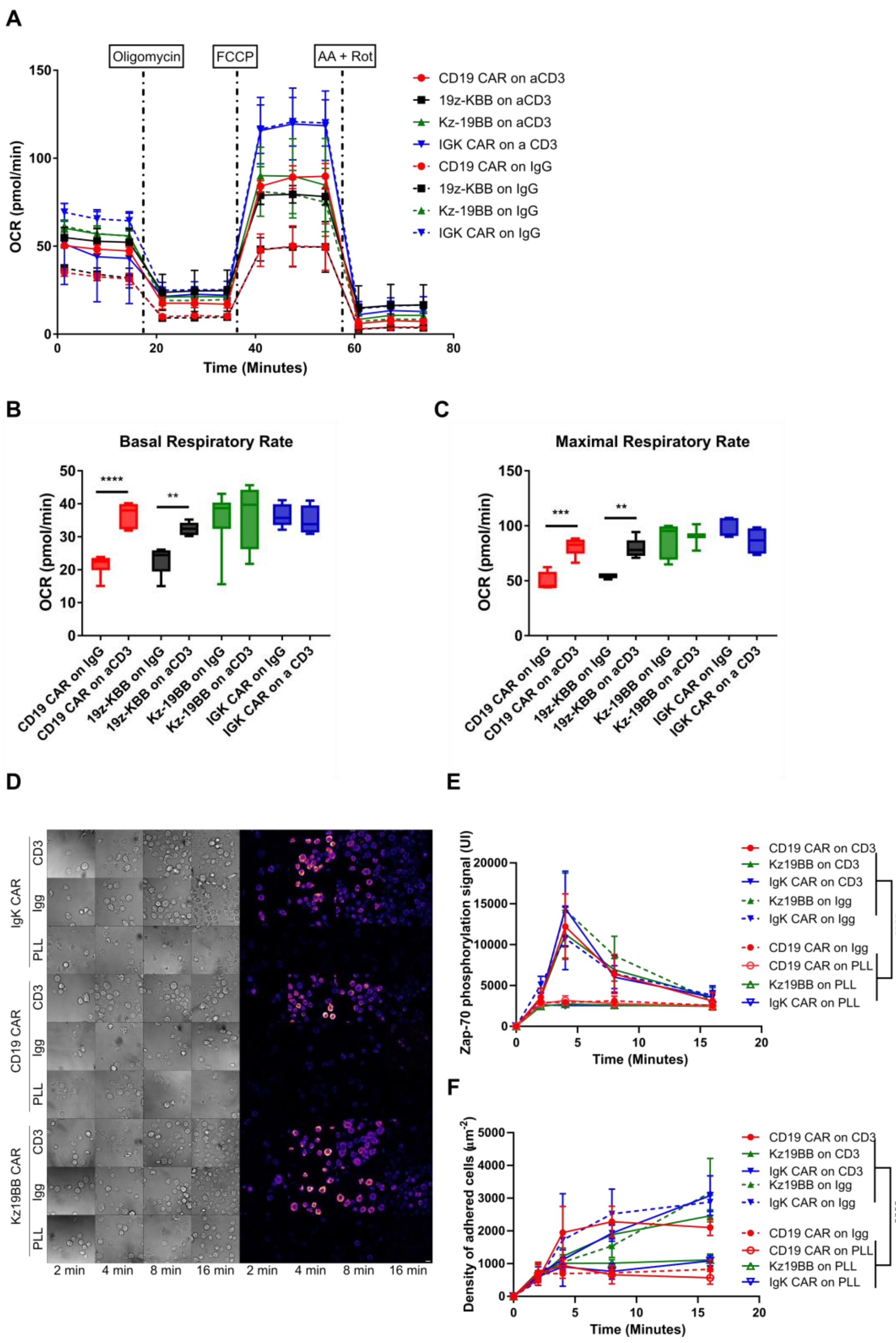

Figure S5: Combinatorial CAR, Kz-19BB maintains IGK CAR properties and is activated in presence of IgG. (A) Primary T cells transfected with CD19 CAR, 19z-KBB, Kz-19BB or IGK CAR coding mRNA were incubated on either anti-CD3 or serum purified IgG coated wells 18 hours after electroporation. Oxygen consumption rate (OCR) was assessed via a Seahorse assay. (B-C) Basal and maximal respiratory rates for each condition. Data represent mean ± S.D. of hexaplicates. Significance

assessed by Student’s t-test. (D) Kz-19BB, CD19 CAR or IGK CAR constructs electroporated T cells were incubated on anti-CD3, IgG or Poly-L-lysine (PLL) for 2, 4, 8 or 16 minutes. Cells were stained with anti-phospho-ZAP70-PE. Representative results for each time point acquired by confocal and corresponding bright field images are shown. Scale bar represents 5µm. (E) ZAP70 phosphorylation signal intensity as analyzed from micrographs. Significance between each construct’s anti-CD3 and IgG response relative to their PLL response was assessed by two-way ANOVA with Tukey's correction (n = 50). (F) Surface adherence properties of each construct when in contact with anti-CD3 and IgG were assessed relative to their PLL response by mixed-effects model (REML). ***P* < 0.01, ****P* < 0.001, ****P<0.0001.

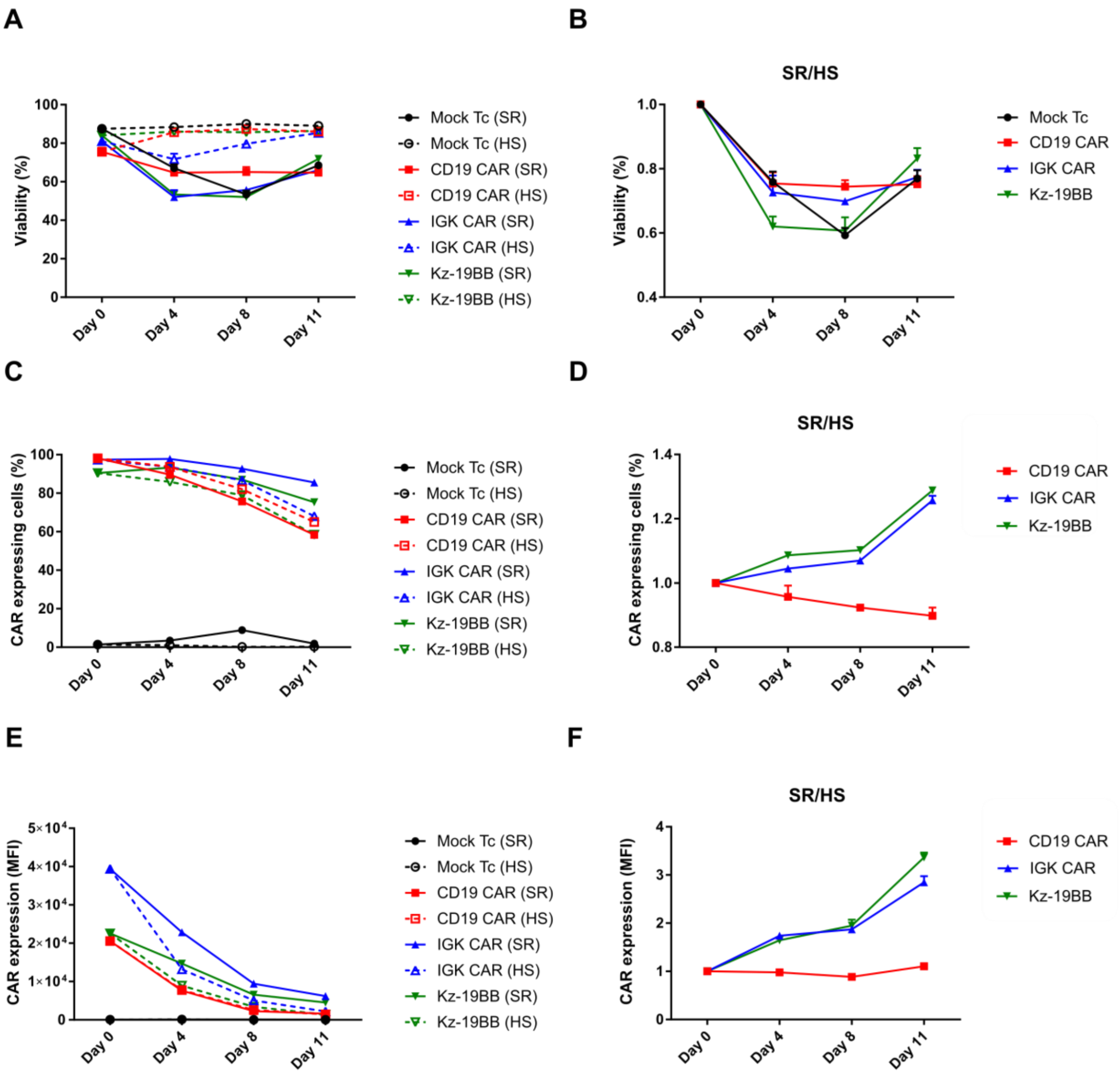

Figure S6: Stable IGK and Kz-19BB CAR-expressing T cells downregulate CAR expression when expanded in human serum. (A-F) Mock and CAR expressing T cells were expanded in either SR (serum replacement) or HS (human serum) containing X-VIVO-15 complete media for 11 days. Cell viability and other properties were evaluated on days 0, 4, 8 and 11. (A-B) Cell viability is monitored with a cell counter utilizing trypan blue exclusion. SR values are divided over HS values to observe the general trend between each expansion method. (C-F) CAR expressions were assesed as before and plotted either as percentage or mean fluorescent intensity (MFI). Similarly, SR values are divided over HS to observe a general trend. Data represent mean ± S.D. of triplicates. Representative data from one of three experiments are shown.

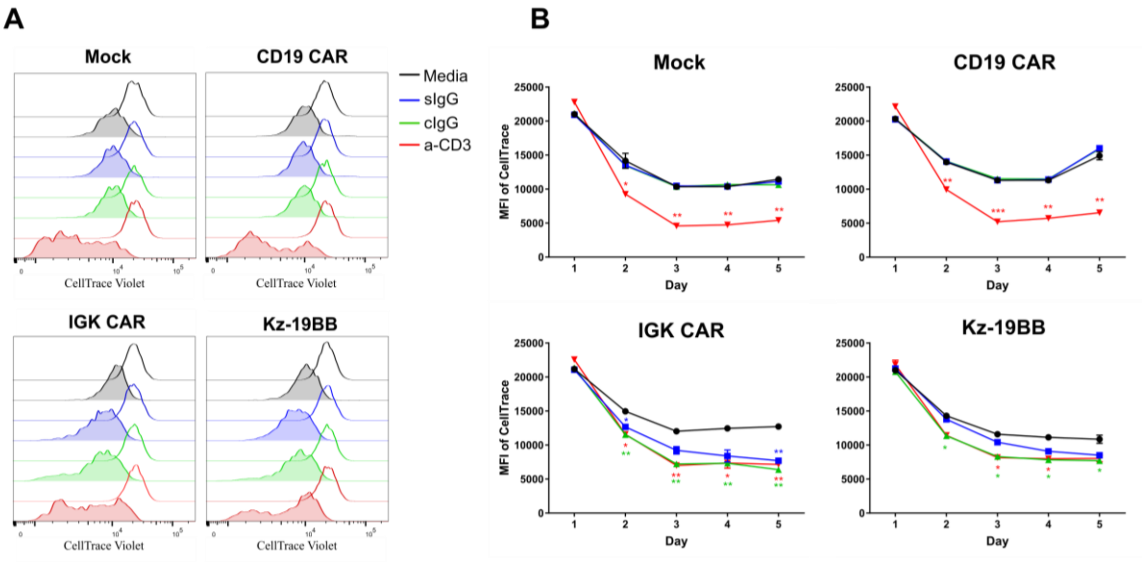

Figure S7: IGK CAR and Kz-19BB mount a proliferative immune response to coated IgG as well as soluble. (A) Mock or construct transduced T cells were cultured in the presence of media or sIgG or on anti-CD19 or IgG coated wells for 5 days. Prior to the experiment T cells were stained with CellTrace Violet and proliferation status was assessed every day through flow cytometry. Representative results show the difference between each group's Day 1 (line) and Day 5 (filled) status. (B) Proliferation assessment of each T cells group over the course of 5 days. Statistical test was performed between each group's media and other conditions by student's t-test. Data represent mean ± S.D. of duplicates. *P < 0.05, **P < 0.01, ***P < 0.001, ****P<0.0001.

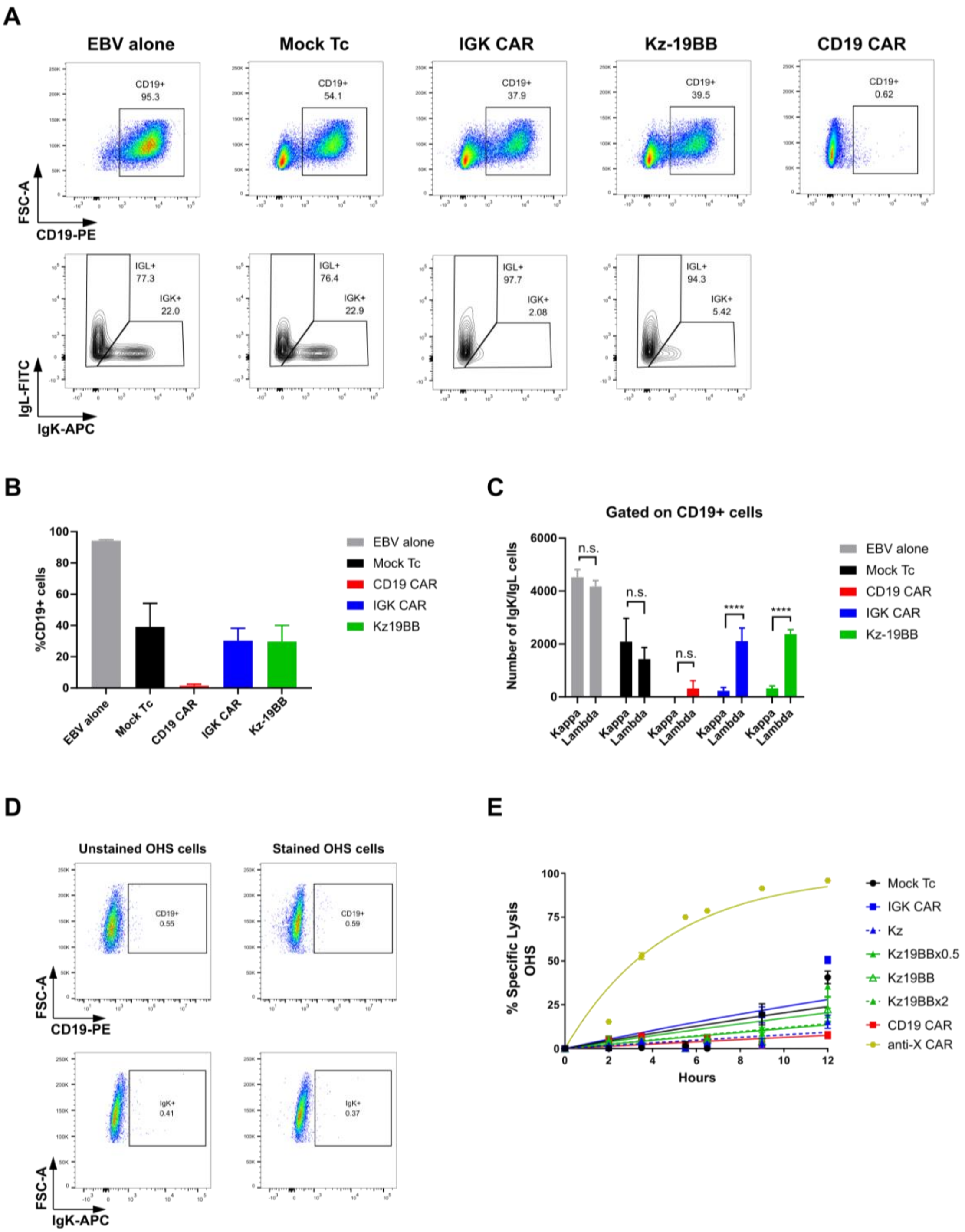

Figure S8: Combinatorial CAR, Kz-19BB selectively eliminates Igκ+ EBV cells in a mixed environment. (A-C) Retrovirally transduced T cells co-cultured for 12 hours with both BL-41 and Granta-519 target cell lines at a ratio of 2:1:1, respectively. After co-culture cells were stained with anti-CD19-PE, anti-Igκ APC and anti-Igλ-FITC. Data represent mean ± S.D. of quadruplicates. Representative data from one of three experiments are shown. Significance was assessed by Student t-test between kappa+ and lambda+ cell numbers. *$P < 0.05$, **$P < 0.01$, ***$P < 0.001$, ****$P<0.0001$. (D) OHS cell line was stained with anti-CD19-PE and anti-Igκ APC. (E) BLI killing assay of Mock and CARs construct electroporated

primary T cells co-cultured with OHS cell line at an E:T ratio of 10:1 for 12 hours. Data represent mean ± S.D. of triplicates. Representative data from one of two experiments are shown.
